# Enhanced recovery after cesarean delivery

**DOI:** 10.12688/f1000research.13895.1

**Published:** 2018-04-27

**Authors:** Unyime Ituk, Ashraf S. Habib

**Affiliations:** 1 Department of Anesthesia, University of Iowa, Iowa City, USA; 2 Department of Anesthesiology, Duke University Medical Center, Durham, USA

**Keywords:** Cesarean Delivery, enhanced recovery after surgery

## Abstract

Enhanced recovery after surgery is a concept initially developed for patients undergoing colorectal surgery but has been adopted by other surgical specialties with similar positive outcomes. The adoption of enhanced recovery after surgery in the obstetric patient population is rapidly gaining popularity. This review highlights perioperative interventions that should be considered in an enhanced recovery after surgery protocol for women undergoing cesarean delivery.

## Introduction

Enhanced recovery after surgery (ERAS) is a concept that combines various evidence-based aspects of perioperative care to accelerate patient recovery. It standardizes perioperative management and achieves a reproducible improvement in the quality of care ^1^. Initial studies on ERAS protocols conducted in colorectal surgery reported a reduction in hospital stay, readmissions, and postoperative complications coupled with improved patient satisfaction ^2– 4^. Since then, there has been widespread adoption of ERAS protocols in other surgical specialties with similar outcomes reported ^5– 8^. The specific components of ERAS protocols differ among surgical specialties and institutions, but the core principles remain the same. These principles involve interventions that span the preoperative, intraoperative, and postoperative periods. It addresses the common reasons that delay patient recovery from surgery and prolong hospital stay such as inadequate analgesia, slow return of bowel function, and delayed ambulation ^9^. There has been slower embrace of the benefits of ERAS in patients undergoing cesarean delivery. However, with increased pressure on maternity services, several centers in Europe have begun implementing ERAS protocols for scheduled cesarean delivery ^10, 11^, and this concept has recently started to gain popularity in the USA. The aim of this review is to highlight evidence-based perioperative interventions that should be considered as part of an ERAS protocol for scheduled cesarean delivery.

## Enhanced recovery pathway for cesarean delivery

### Why enhanced recovery for cesarean delivery?

The cesarean delivery rate in the United States is about 32% of all births, with over 1.27 million procedures performed annually ^12^. The majority of women undergoing cesarean delivery are young and healthy and therefore have the potential for rapid recovery following delivery. Furthermore, being able to care for their newborn provides an added motivation to return to normal physiological function. A study on early discharge following uncomplicated cesarean delivery that pre-dates the concept of ERAS reported higher maternal satisfaction in the early discharge group compared to women in a routine care group ^13^.

There are already many aspects of current routine perioperative care of the patient undergoing a cesarean delivery that are consistent with components of ERAS. A survey of obstetric anesthesiologists in the UK conducted in 2013 showed that the majority of respondents supported the concept of ERAS for cesarean delivery and most were considering or were in the process of implementing an ERAS protocol at their institutions ^10^. A similar survey of 36 academic maternity units in the UK conducted in 2015 reported that 50% of respondents had implemented an ERAS protocol and 30% had plans to introduce one ^14^. ****

### Proposed components for enhanced recovery after cesarean delivery

**Figure 1.**
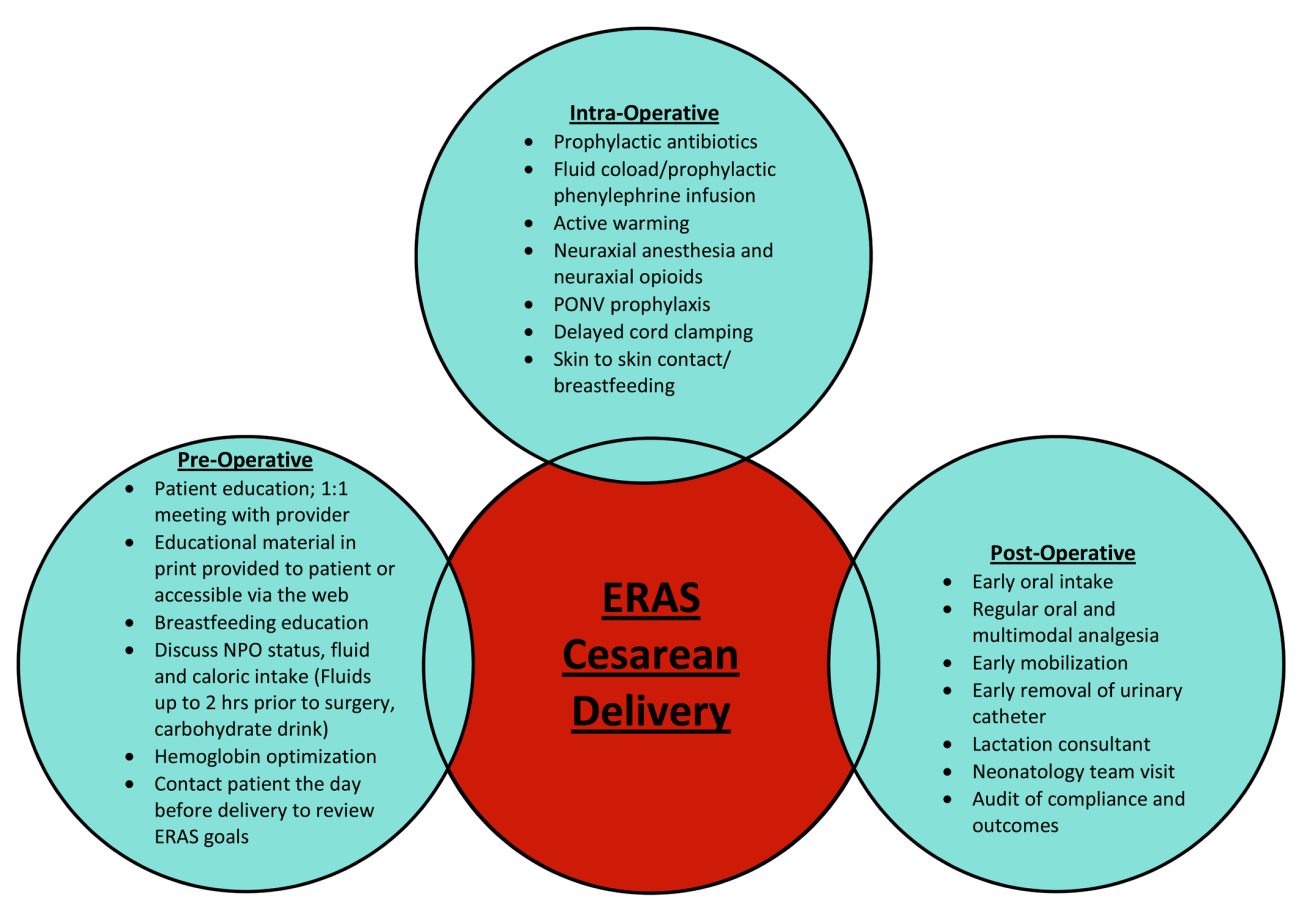
Components of Enhanced Recovery Protocol for Cesarean Delivery. ERAS, enhanced recovery after surgery; NPO, *nil per os* (nothing by mouth); PONV, postoperative nausea and vomiting.

The principles of enhanced recovery cover the entire perioperative care pathway and component interventions occur during the preoperative, intraoperative, and postoperative phases of care ^15^ ( Figure 1).

## Preoperative preparation

### Patient education

Patient education and counselling and a shared decision-making model are required for the successful implementation of an ERAS program. Studies on ERAS implementation in various surgical specialties have reiterated the need for active participation of the patient in the recovery process and its positive impact on patient outcomes ^16^. In a recent study focused on patient education to enhance recovery in colorectal surgery, the authors reported that patients wanted to be proactively involved in their recovery process ^17^. Active patient engagement can be achieved by a comprehensive and timely preoperative education that includes provision of internet-accessible or take-home educational materials allowing patients to be acquainted with the ERAS concepts. Patient education should include information on the procedure and what to expect during surgery, a pain management plan, and goals for early feeding and mobilization. Information should also be provided on breastfeeding, including lactation support services available, length of stay, and the criteria for discharge. Patients can be given a checklist with actions and goals which they can use to keep track of their progress in the recovery process ^18^.

### Nil per os status, preoperative fluid, and caloric intake

Traditionally, patients have been told to fast from midnight before surgery to reduce the risk of pulmonary aspiration. Ultrasonography studies have demonstrated that gastric emptying is normal during pregnancy and is slowed only with the onset of labor ^19, 20^. The current practice guidelines for obstetric anesthesia from the American Society of Anesthesiologists (ASA) recommend six- to eight-hour fasting for solids and clear oral fluid intake up to two hours before the induction of anesthesia ^21^. The intake of a high-caloric carbohydrate drink up to two hours before surgery has been shown to reduce preoperative thirst, hunger, and anxiety in patients undergoing abdominal surgery ^22^. It has also been associated with a reduction in insulin resistance and a higher anabolic state postoperatively ^23, 24^. ****

### Preoperative hemoglobin optimization

There are inadequate data on the prevalence of iron deficiency anemia in pregnant women in the United States. The National Health and Nutrition Examination Survey (NHANES) from 1999 to 2006 estimated the prevalence of iron deficiency in pregnant women as 18.6% ^25^. Most women presenting for prenatal care are routinely screened for anemia. However, there are differing opinions among government health agencies and professional associations on the benefits of routine screening and iron supplementation in asymptomatic pregnant women. The Centers for Disease Control and Prevention (CDC) recommend screening for anemia and initiating low-dose iron supplementation for all pregnant women at the first prenatal care visit ^26^. The American College of Obstetricians and Gynecologists (ACOG) also recommends anemia screening for all pregnant women but treating only those with anemia with supplemental iron ^27^. On the other hand, the United States Preventive Services Task Force (USPSTF) and the American Academy of Family Physicians (AAFP) conclude that the current evidence is insufficient to recommend for or against routine screening and iron supplementation to prevent adverse maternal and neonatal outcomes ^25^. At our institutions, pregnant women are routinely screened for anemia and are referred to an anemia clinic for optimization of hemoglobin if anemic or if they have an increased risk of obstetric hemorrhage. Furthermore, preoperative anemia is a significant predictor of severe postpartum anemia, which has been linked to various morbidities such as depression and fatigue ^28^.

## Intraoperative care

### Prophylactic antibiotics

Cesarean delivery increases the risk of infection and its related morbidity 5 to 20-fold compared to vaginal delivery ^29^. Infectious complications lead to hospital readmissions ^30^ and a significant increase in length of hospital stay. There is compelling evidence that prophylactic antibiotics should be administered to all women undergoing cesarean delivery ^29^. Traditionally, prophylactic antibiotics have been withheld until cord clamping owing to concern of neonatal exposure to antibiotics. There is, however, conclusive evidence that prophylactic antibiotics administered within 60 minutes before skin incision significantly reduce the incidence of maternal postpartum infection compared to administration after cord clamping ^31, 32^. The current recommendation is a single dose of a broad-spectrum antibiotic in the non-laboring patient prior to skin incision ^33^.

### Thromboprophylaxis

Pneumatic compression devices are recommended for all women undergoing cesarean delivery and not already receiving pharmacologic thromboprophylaxis ^34^. The compression devices should be continued until the patient is fully ambulatory. In women with one or more additional risk factors, pharmacological thromboprophylaxis is recommended ^35^.

### Fluids and blood pressure management

One of the core principles of ERAS is the maintenance of a normal fluid balance. In the general surgical population, goal-directed fluid therapy based on physiologic endpoints has been shown to reduce perioperative complications and length of stay ^36^. The benefits of goal-directed fluid therapy as part of an ERAS protocol are less clear and have generated much debate among clinicians ^37– 39^. The usefulness of goal-directed fluid therapy has not been investigated in the cesarean delivery population but might be valuable given the likely different hydration status of women presenting for cesarean delivery.

Hypotension occurs commonly in women undergoing cesarean delivery under spinal anesthesia, and it can be detrimental to the mother and the fetus. Hypotension can trigger intraoperative nausea and vomiting (IONV) in the mother and decrease uteroplacental blood flow, which impairs fetal oxygenation. Both fluids and vasopressors have been used to counteract spinal anesthesia-induced hypotension. Fluid loading strategies alone have limited efficacy in reducing the incidence of hypotension ^40^. However, when used in conjunction with a prophylactic phenylephrine infusion, a rapid crystalloid coload of 2 L was associated with a significant reduction in the incidence of hypotension compared to maintenance fluid administration ^41^.

Given that the etiology of spinal-induced hypotension is mainly related to peripheral vasodilatation, vasopressors are the mainstay for the management of hypotension. Phenylephrine is currently considered the vasopressor of choice for the management of maternal hypotension induced by neuraxial anesthesia, given its favorable fetal acid–base status and lower incidence of IONV when compared with ephedrine ^42– 44^. A prophylactic infusion is more effective at reducing the number of hypotensive events as well as decreasing the incidence of nausea and vomiting compared to rescue treatment of established hypotension with phenylephrine boluses ^45^. Therefore, the recommended strategy as part of an ERAS protocol would be to use a prophylactic phenylephrine infusion initiated at 50 mcg/minute in conjunction with a rapid crystalloid coload of up to 2 L. A low-dose norepinephrine infusion has been investigated as an alternative to phenylephrine in managing hypotension during cesarean delivery. Studies suggest similar efficacy in maintaining blood pressure with a higher heart rate and cardiac output compared to phenylephrine ^46– 48^.

### Temperature management

Maintaining perioperative normothermia in the general surgical population reduces the risk of postoperative wound infection, coagulopathy, blood loss, and transfusion requirement ^49, 50^. The incidence of hypothermia in women undergoing cesarean delivery under spinal anesthesia is estimated to be >60%. Temperature autoregulation is impaired during spinal anesthesia by the inhibition of vasomotor and shivering responses and a redistribution of heat from the core to the peripheral tissues. Hypothermia associated with spinal anesthesia might be under-appreciated ^51^. A recent study demonstrated a rapid drop in intestinal temperature by a mean of 1.3 °C during cesarean delivery under spinal anesthesia ^52^. The median time to the lowest intestinal temperature was one hour after the initiation of spinal anesthesia, and temperature continued to fall in the majority of patients even after completion of the procedure. It took a median of 4.5 hours for intestinal temperature to recover to baseline, and, in 29% of patients, the temperature did not return to baseline during the 8-hour duration of the study. However, patients in this study were not actively warmed. Perioperative hypothermia can be a cause for delayed discharge from the post anesthesia care unit (PACU), which has been correlated with an increased length of stay during implementation of ERAS protocols for cesarean delivery ^53^.

Hypothermia-related adverse outcomes in women undergoing cesarean delivery have not been adequately examined ^51^. A meta-analysis of 13 randomized trials by Sultan *et al*. reported that active warming in women undergoing elective cesarean delivery under spinal anesthesia reduced the maximum fall in temperature and decreased the incidence of hypothermia and shivering when compared with controls not actively warmed ^54^. Thermal comfort was also improved in patients who had active warming, together with reduced neonatal hypothermia and improved umbilical artery cord pH. Maintaining normothermia can also help facilitate early maternal bonding with the newborn.

The best strategy for active warming is unclear. Most strategies have limited efficacy in isolation, and a combination of preoperative and intraoperative forced air warming with warmed intravenous fluids may be more effective ^51^ and should be implemented as part of all ERAS protocols.

### Neuraxial anesthesia including neuraxial opioids for analgesia

Neuraxial anesthesia (mainly spinal anesthesia) is the anesthetic technique of choice for elective cesarean delivery ^55– 57^. Neuraxial anesthesia decreases the hypothalamo-pituitary response to surgical stress and has been shown to reduce the duration of postoperative ileus in the general surgical population ^58^. Neuraxial anesthesia also allows the woman to witness the birth of her child, allows for early skin-to-skin contact with the newborn, and facilitates the presence of a support person in the operating room.

Opioids are usually added to local anesthetic mixture because they improve intraoperative anesthesia, prolong its duration, decrease local anesthesia requirements, and provide postoperative analgesia ^59^. A lipophilic fast-onset and short-acting opioid such as fentanyl or sufentanil is added for intraoperative effects, and a hydrophilic opioid such as morphine with a prolonged duration of action is added for postoperative analgesia. Neuraxial morphine provides superior analgesia compared to systemic opioid administration. Studies suggest a ceiling in analgesic effect with a dose-related increase in opioid-related side effects, including nausea, vomiting, and pruritus. In a recent meta-analysis, Sultan *et al*. reported that the odds of nausea or vomiting (OR 0.44 [95% CI 0.27–0.73]) and pruritus (OR 0.34 [95% CI 0.20–0.59]) were lower with smaller doses of intrathecal morphine (50–100 mcg) than with higher doses (>100 mcg) ^60^, but the time to first request of analgesia was longer by an average of 4.5 hours with the higher doses, with no difference in total postoperative analgesic consumption.

There is wide variability in analgesic requirements after cesarean delivery, and tests such as quantitative sensory testing, hyperalgesia testing, response to local anesthesia skin infiltration, and psychometric evaluation may help identify patients with higher postoperative analgesic requirements ^61^. However, studies investigating postoperative analgesia use based on the results of these predictive tests are lacking. The preference of the patient for more analgesia versus side effects should also be considered. A study by Carvalho *et al*. reported that women who preferentially chose a larger intrathecal morphine dose correctly anticipated greater postoperative opioid requirement and more pain compared with women who chose the smaller dose ^62^.

### Postoperative nausea and vomiting prophylaxis

Postoperative nausea and vomiting (PONV) can delay early oral intake, a key objective of ERAS. PONV occurs frequently after cesarean delivery, especially in parturients who received neuraxial opioids ^63^. The etiology of PONV is multifactorial, and therefore a multifaceted approach for prophylaxis is needed. Combination of anti-emetic agents is more effective in the management of PONV compared to monotherapy; however, studies investigating combination anti-emetic therapy in the obstetric patient population are scarce ^64, 65^. Use of combination therapy of non-sedating agents such as ondansetron with dexamethasone should be an integral part of an ERAS protocol. Droperidol, an antidopaminergic agent, is also effective for PONV prophylaxis ^65^. There is, however, a “black box” warning by the Food and Drug Administration (FDA) because of risk of torsade de pointes due to QT prolongation, and this has limited its use in the USA ^66^.

IONV is common during cesarean delivery under spinal anesthesia ^63^. Avoidance of hypotension with a prophylactic phenylephrine infusion, administration of metoclopramide, and avoidance of uterine exteriorization and fluid irrigation have been associated with a reduced incidence of IONV ^64^.

### Delayed cord clamping

Delay in clamping of the umbilical cord for at least 30 seconds was initially recommended in preterm newborns because it is associated with a reduction in risk of intraventricular hemorrhage, an increase in hematocrit, and a decrease in need for volume resuscitation ^67, 68^. However, current data suggest that it may also be beneficial in term infants without evidence of significant harm. A meta-analysis of 15 trials by McDonald *et al*. reported that delayed cord clamping was associated with higher hemoglobin concentration and iron reserves up to six months after birth compared to early clamping ^69^. There was, however, a higher risk of jaundice requiring phototherapy in infants who had delayed cord clamping. The current recommendation from ACOG is delayed cord clamping in vigorous term and preterm infants for at least 30–60 seconds after birth ^70^.

### Skin to skin

There are reported benefits for both the newborn and the mother of early skin-to-skin contact. Early skin to skin has been associated with increased rates and duration of breastfeeding ^71, 72^ and a decrease in maternal anxiety and postpartum depression ^73^. However, maternal intent to breastfeed may be associated with an increased length of hospital stay ^53^. If an ERAS protocol for cesarean delivery is to be successful, steps should be taken to support the early initiation of breastfeeding. A concept termed “natural or gentle” cesarean delivery developed about a decade ago seeks to modify some aspects of cesarean delivery so that the woman can have a “natural” experience comparable to a vaginal birth ^74^. These modifications include using a transparent surgical drape, allowing the mother and her partner to witness the birth, and initiating immediate skin-to-skin contact and breastfeeding after birth. A randomized trial comparing the “natural” cesarean delivery to a traditional cesarean delivery reported a significantly greater rating of birth experience and higher breastfeeding in the “natural” cesarean delivery group ^75^.

### Oxytocin management

A prophylactic low-dose oxytocin infusion (15–18 U/hour) should be commenced to prevent postpartum hemorrhage ^76^. A low dose reduces the occurrence of adverse effects such as hypotension and myocardial ischemia ^77^. Carbetocin, a long-acting oxytocin receptor agonist available in Canada and Europe, can also be used as a first-line prophylactic uterotonic instead of oxytocin ^78^.

## Postoperative care

### Early oral intake

Traditionally, oral intake has been delayed after abdominal surgery until the return of bowel function is confirmed by bowel sounds or passage of flatus or stools. This is contrary to the current evidence indicating that early oral intake promotes the return of bowel function and early ambulation, decreases the risk of sepsis, reduces the time to breastfeeding, and shortens the length of stay ^79– 81^.

### Regular oral and multimodal analgesia

Provision of adequate postoperative analgesia is an integral component of ERAS protocols, and it assumes even greater importance in women undergoing cesarean delivery. Suboptimal analgesia is associated with delayed functional recovery, delayed mobilization which could increase the risk of thromboembolic complications, poor maternal bonding with the newborn, breastfeeding difficulties, and an increase in the risk of persistent pain and postpartum depression ^82, 83^. There are multiple complex factors that contribute to postoperative pain, with significant inter-individual variability in pain perception. ERAS protocols recommend a multimodal analgesic regimen using a combination of drugs with different mechanisms of action with the goal of optimizing analgesia, minimizing side effects, and providing opioid sparing ^16^. This can be achieved through the combination of neuraxial opioid analgesia, oral analgesia, and peripheral nerve blockade. Neuraxial morphine was discussed earlier in this review and is considered the gold standard for post cesarean analgesia. Recommended dosing is 100–150 mcg intrathecal and 3 mg epidural ^84, 85^. However, supplemental opioid-sparing analgesics are required to optimize the quality of postoperative analgesia and decrease the need for additional rescue oral or intravenous opioids.

***Acetaminophen and non-steroidal anti-inflammatory drugs***. Acetaminophen has an opioid-sparing effect and provides analgesia with minimal adverse effects or secretion in breast milk ^86– 88^. An acetaminophen–opioid combination is commonly prescribed for breakthrough pain because of the synergistic effect between the two agents, but scheduled acetaminophen with as-needed opioids is recommended. In a study comparing opioid use in patients on scheduled acetaminophen with as-needed opioids to patients on as-needed acetaminophen plus opioids, the cumulative opioid use was reported to be significantly reduced in patients receiving scheduled acetaminophen ^89^.

Nonsteroidal anti-inflammatory drugs (NSAIDs) have an opioid-sparing effect of up to 50% ^90^. Acetaminophen and NSAIDs have an additive analgesic effect and, unless contraindicated, both drugs should be routinely given on a scheduled rather than a *pro re nata* basis after cesarean delivery ^91^.

***Nerve blocks and wound infiltration***. Transversus abdominis plane (TAP) block, local anesthetic wound infiltration, and, recently, quadratus lumborum (QL) block have all been described as adjuvant techniques for analgesia after cesarean delivery. TAP blocks improve postoperative analgesia after cesarean delivery in patients who did not receive spinal morphine but not in those who received intrathecal morphine ^92, 93^.

There are limited data on the efficacy of local anesthetic wound infiltration in women undergoing cesarean delivery. Local infiltration of NSAIDs might also provide benefit, but the value of wound infiltration in patients receiving spinal morphine with a multimodal analgesic regimen is unclear ^94^. A recent study comparing spinal morphine to a continuous infusion of ropivacaine into the surgical wound suggested improved analgesia in both groups compared to control, but rescue opioid consumption was lower with spinal morphine ^95^.

Recent studies have shown that the QL block provides effective analgesia after cesarean delivery and reduces opioid consumption ^96, 97^. However, the analgesic efficacy of QL block in patients who receive spinal morphine has not been investigated.

In summary, local anesthetic techniques have limited efficacy when used in conjunction with neuraxial morphine but should be considered in patients who do not receive neuraxial morphine or when high postoperative analgesic needs are anticipated. Furthermore, it is not clear if techniques using long-acting liposomal local anesthetics might confer benefit in patients receiving neuraxial morphine.

### Early mobilization

Early mobilization improves pulmonary function and tissue oxygenation, improves insulin resistance, reduces risk of thromboembolism, and shortens length of stay ^98^. Effective postoperative analgesia is a key factor in facilitating early postoperative mobilization. Mobilization goals after cesarean delivery should be discussed during the preoperative patient education.

### Early removal of urinary catheter

It is recommended that urinary catheters are removed within 24 hours in ERAS protocols. There are few data on the timing of urinary catheter removal in women who have cesarean delivery under spinal anesthesia. In a published audit of an ERAS protocol for cesarean delivery, urinary catheters were removed 7 hours after the procedure to facilitate early ambulation with no complications reported ^99^.

### Post discharge

Prior to discharge, it must be ensured that the patient has access to a reliable means of communication with the labor and delivery unit, is given the number to call, and knows who to contact if there are any concerns. The patient must be contacted within 24 hours after discharge to assess the wellbeing of the mother and the newborn and to address any questions or concerns.

## Barriers to implementation

The potential barriers to successful implementation of an ERAS protocol for cesarean delivery include the discomfort providers feel with change in practice, allocation of resources especially for patient education, post discharge follow up, and the lack of dedicated operating rooms for scheduled cesarean deliveries ^15^. Goals should be set and targets audited regularly to identify compliance and opportunities for improvement. Coordination with the neonatology team and lactation consultants is also crucial to avoid delays in discharge due to issues related to neonatal tests and evaluation or breastfeeding education.

## Conclusion

An enhanced recovery program for cesarean delivery should consist of the best evidence in perioperative care of the parturient. There is wide variability in components of published ERAS protocols for cesarean delivery. Future studies on developing and evaluating the impact of various components are needed.
